# How much gaming is too much? An analysis based on psychological distress

**DOI:** 10.1556/2006.2024.00036

**Published:** 2024-07-18

**Authors:** Dana Katz, Zsolt Horváth, Halley M. Pontes, Patrik Koncz, Zsolt Demetrovics, Orsolya Király

**Affiliations:** 1 Doctoral School of Psychology, ELTE Eötvös Loránd University, Budapest, Hungary; 2 Institute of Psychology, ELTE Eötvös Loránd University, Budapest, Hungary; 3 School of Psychological Sciences, Birkbeck, University of London, London, United Kingdom; 4 Centre of Excellence in Responsible Gaming, University of Gibraltar, Gibraltar, Gibraltar; 5 College of Education, Psychology and Social Work, Flinders University, Adelaide, Australia

**Keywords:** gaming disorder, gaming time, psychological distress, latent profile analysis

## Abstract

**Background:**

While there are calls to restrict the time spent on gaming because it is seen as problematic and potentially leading to gaming disorder (GD), there is conflicting evidence about this issue. We explored the association between the average weekly time spent gaming and reported GD symptoms. Additionally, Latent Profile Analysis was employed to investigate how time spent gaming relates to variables representing psychological distress (PD), such as satisfaction with life, symptoms of depression, and perceived stress.

**Methods:**

Data were collected using surveys with a large sample of highly engaged gamers (*N* = 14,740; Mage = 24.14 years, SDage = 7.0, 89.3% males).

**Results:**

We observed a positive, close to linear association between time spent gaming and GD symptoms. Groups at risk of GD played for about 42 h (SD = 19) on average, according to the American Psychiatric Association and World Health Organization frameworks. Furthermore, we identified four profiles representing varying levels of PD. Gamers reporting very high levels of PD (4.2% of the sample) played for 33 h per week on average. Remarkably, a substantial percentage of the sample (41.9%) showed no PD despite playing for 26 h per week.

**Conclusion:**

The association between gaming time and PD is complex as even prolonged time spent gaming can be unproblematic for many gamers.

## Introduction

The popularity of video gaming as a leisure activity is increasing continuously and steadily across all age groups and genders (Entertainment Software Association, 2021). In 2022, the gaming market generated approximately $184.4 billion US dollars, and more than 3.0 billion players were registered, most of them playing on mobile phones (Newzoo, 2022). While for the majority, playing video games is a beneficial activity that enriches people's personal and social lives (Demetrovics et al., 2011), for a minority, it becomes problematic, ultimately leading to various negative outcomes (Byeon et al., 2022; Zhou et al., 2020).

Initially, the American Psychiatric Association (APA) tentatively indicated that what it termed as Internet gaming disorder (IGD) warranted further study. The association included this disorder in the 5th revision of the Diagnostic and Statistical Manual of Mental Disorders in 2013 (DSM-5; American Psychiatric Association, 2013), and it retains this status in the recent DSM-5-TR (DSM-5; American Psychiatric Association, 2022). The criteria for IGD include preoccupation, withdrawal, tolerance, loss of control, loss of interest, continued use despite negative consequences, deception, escapism, and negative consequences. Subsequently, in 2019, the World Health Organization (WHO) officially recognized what it called GD as a formal psychiatric disorder in the 11th revision of the International Classification of Diseases (ICD-11; World Health Organization, 2019). The organization defined it as “*persistent or recurrent gaming behavior which results in marked distress or significant impairment in personal, family, social, educational, occupational, or other important areas of functioning*.” [c. 6C51]. The GD criteria in the ICD-11 include loss of control, loss of interest, and continued use despite negative consequences. To date, experts still agree on these criteria (Calvo et al., 2021; Király, Potenza, & Demetrovics, 2022c).

Apart from differences in criteria, each of the diagnostic frameworks also adopts a distinct approach. While DSM-5 adheres to a polythetic approach, meaning that an individual must meet a certain number of criteria, the ICD-11 follows a monothetic approach, meaning that an individual must meet the set of necessary and sufficient criteria precisely, providing a more rigorous diagnosis. Interestingly, in the wake of major criticism (Griffiths et al., 2016; Király, Griffiths, & Demetrovics, 2015), the preoccupation criterion for GD in the ICD-11 was omitted. Although it appears in the criteria for IGD in the DSM-5, the definition itself does not quantify a gaming time threshold. For the sake of consistency, we will use the term GD regardless of the diagnostic framework to which we refer (i.e., DSM-5 [IGD] or ICD-11 [GD]), as it symbolizes the same phenomenon and is now used as the official term.

Scholars have been trying to identify individuals at risk of developing GD and detect the factors involved in its incidence and persistence. To date, they have indicated that being male and of a younger age (Stevens, Dorstyn, Delfabbro, & King, 2021) and having psychological vulnerabilities such as anxiety and low self-esteem (Gao, Wang, & Dong, 2022) are some of the factors underpinning GD. Video games are immersive and enticing, prompting people to spend more time on them. Indeed, researchers have established a positive association between the amount of time spent playing video games and the persistence of GD (Bargeron & Hormes, 2017; Pontes, Király, Demetrovics, & Griffiths, 2014; Pontes & Griffiths, 2016). However, in most studies, this association appeared to be weak or moderate (e.g., Laconi, Pirès, & Chabrol, 2017; Lemmens, Valkenburg, & Gentile, 2015), depending on factors such as the type of gaming device (Montag, Schivinski, & Pontes, 2021), the genre (Rehbein, King, Staudt, Hayer, & Rumpf, 2021), and the design elements (Király, Koncz, Griffith, & Demetrovics, 2023). These results suggest that prolonged time spent gaming is problematic only in certain cases.

Thus, the issue of the time spent gaming remains somewhat controversial. Although excessive gaming can be viewed as a severe risk factor for developing GD, it has been long established that increased time spent gaming might not necessarily be a major aspect of the addictive process (Charlton, 2002; Charlton & Danforth, 2007). Nevertheless, driven by the notion that the amount of time is the primary problem, numerous scientific and legislative efforts have been devoted to reducing the amount of time spent gaming (Colder Carras, Stavropoulos, Motti-Stefanidi, Labrique, & Griffiths, 2021). A recent example occurred in mainland China, where the government enacted laws to limit the time spent gaming among under-aged individuals to only one hour on Fridays, weekend days, and public holidays between 8 PM and 9 PM. These laws raised questions about whether such decisions were responsible or actions that criminalized an activity that is harmless and recreational for most (Colder Carras et al., 2021; Király, Browne, & Demetrovics, 2022a). Many maintained that such measures were ineffective and would not curb GD (Zendle et al., 2023). Others argued that a better approach would be to provide factual information through education and counseling and develop measures for early detection of problematic or addictive usage patterns to support gamers and their families (Czakó et al., 2023).

Apart from determining to what extent the time spent gaming might be problematic, understanding its complex relationships with other factors may be particularly important. Based on the theoretical grounds set by the Interaction of the Person-Affect-Cognition-Execution model (I-PACE), predisposing conditions such as vulnerability to stress or depression may prompt deficits in behavioral control and nurture addictive patterns characterized by uncontrollable amounts of time gaming (Brand, Young, Laier, Wölfling, & Potenza, 2016, 2019). Such addictive patterns are either developed in early stages due to gratification or later on as compensation. In this context, recent results by Strojny, Żuber, and Strojny (2024) have highlighted depression as a moderator of the dosage effect, meaning the association between the amount of time spent gaming and GD symptoms. They attributed the role of depression to amplifying gratification and conceivably contributing to the development of GD. Therefore, although there is a possible link between gaming time and pathology, it is imperative to examine the role of the amount of time in this relationship more rigorously. This is paramount as some studies have established that prolonged gaming time has a positive correlation with well-being gradually (Johannes, Vuorre, & Przybylski, 2021). Furthermore, even 7–10 h or more of time spent gaming per week resulted in better mental health outcomes when compared to no gaming at all (Jones, Scholes, Johnson, Katsikitis, & Carras, 2014).

In an attempt to answer one of the most frequent questions regarding GD, namely, “*How much gaming time is too much?*” Pontes, Schivinski, Kannen, and Montag (2022) conducted an online survey involving a large sample of video gamers. They found that the risk of developing GD was associated with 35 or 40 h of gaming a week on average, according to the APA and WHO frameworks, respectively. These numbers are considerably higher than those specified in the DSM-5 (i.e., 30 h per week or more) (DSM-5; American Psychiatric Association, 2013; 2022). Furthermore, the authors also found that gamers who did not exhibit a single GD symptom reported playing about 20 h a week on average. Hence, these findings imply that playing video games for an amount of time equivalent to a half-time job is not necessarily associated with negative consequences such as conflicts with significant others or the neglect of personal responsibilities. However, given that the view of gaming time as a factor determining the level of GD is still prevalent, especially among the general population, a thorough insight into the interaction between time spent gaming and psychological distress (PD) may help to understand its role.

To accomplish this goal, we conducted a study with two aims. First, we investigated the association between the time spent gaming and GD symptoms according to the APA and WHO diagnostic frameworks. In this case, we wanted to determine whether the findings of Pontes et al. (2022) could be replicated among a large sample of highly engaged Hungarian gamers. Our second aim was to broaden the work of Pontes and colleagues by exploring the association between the time spent gaming and variables representing PD. To delineate the spectrum of PD, we included both well-being measures, such as satisfaction with life, and ill-being measures, such as symptoms of depression and perceived stress, to obtain a more nuanced representation of the situation. The correlations among the three variables ranged from −0.454 to 0.649, supporting the internal consistency of the composite score (for more details, see Supplementary Table S1). Gamers were divided into homogeneous groups based on varying levels of PD, and self-reported weekly time spent gaming was averaged for each group to determine what amount of time characterizes those experiencing considerable PD.

## Methods

### Study's design and ethics

This cross-sectional study used an existing data set from a study that has already been published (Király et al., 2022). However, the conceptualization, goal, analyses, and results of the present study are novel and dissimilar from the previous research. Furthermore, the data analyzed in the present study are entirely different from the data that Pontes and colleagues used in their 2022 paper.

### Participants and procedure

Data were collected during March and April 2020 (the first quarantine period due to the COVID-19 pandemic). An online survey was created and administered in Qualtrics (https://www.qualtrics.com) to collect data from highly engaged video gamers, defined as those who spent at least 20 h a week engaged in gaming. The online gaming magazine GameStar.hu, which is very popular among Hungarian-speaking gamers living in Hungary and surrounding countries such as Romania, Slovakia, Serbia, and Ukraine, promoted the survey.

To increase participation, we offered those who completed the survey the opportunity to enter a drawing to win shopping vouchers of various amounts. Before starting the survey, participants were informed about the aims of the study and assured anonymity and confidentiality. They provided their informed consent electronically by ticking a box if they agreed to participate. Under-aged participants between the ages of 14 and 17 had to tick an additional box to indicate whether parental permission had been obtained. All data cleaning procedures and analyses were conducted using SPSS version 28 (IBM Corp., 2021) and Mplus 8 (Muthén & Muthén, 1998–2017). The data-cleaning process yielded 14,740 valid responses. For more details, see Király et al. (2022).

### Measures

#### Socio-demographic variables

Socio-demographic information was collected, including the participants' age, gender, marital status, education (i.e., number of years completed), and current work and study status.

#### Gaming disorder symptoms

##### The American Psychiatric Association's (APA) framework

Based on the APA framework, the Ten-Item Internet Gaming Disorder Test (IGDT-10) was used to assess GD symptoms (Király et al., 2017, 2019). The scale was developed using the diagnostic criteria of the IGD proposed in the DSM-5 (DSM-5; American Psychiatric Association, 2013) and Petry et al.’s (2014) suggestions. Responses to items such as ‘*Have you ever unsuccessfully tried to reduce the time spent on gaming?*’ were made on a 3-point scale (0 = ‘never,’ 1 = ‘sometimes,’ and 2 = ‘often’). During the analyses, items were recoded into ‘yes’ (1) and ‘no’ (0) such that responses ‘never’ and ‘sometimes’ were coded as 0, and ‘often’ was coded as 1, in accordance with the DSM-5 approach (i.e., fulfilling a criterion or not). Given that items 9 and 10 belonged to the same criterion (i.e., ‘*Has jeopardized or lost a significant relationship, job, or educational or career opportunity because of participation in Internet games’*), they were combined so that ‘often’ on one or both items was recoded as 1 point. Scores were summed and ranged from 0 to 9, indicating the number of GD symptoms exhibited according to the APA framework. The composite reliability of the scale with the nine binary items was ω = 0.88 in the present study. During the analyses, GD scores were used in two forms. To compare the groups, we conducted ANOVAs in which we merged gamers exhibiting seven (*n* = 40), eight (*n* = 15), and nine (*n* = 6) GD symptoms into one group, combining these scores (i.e., ≥7 [7, 8, 9 GD scores merged]); see Table 1). We adopted this method due to the small number of respondents in each of these categories. For the boxplots, GD categories were calculated so that scores of 0–4 indicated no risk of GD, and scores of 5 or more indicated the risk of developing GD, in accordance with the DSM-5 recommendation (DSM-5; American Psychiatric Association, 2013).

**Table 1.** Descriptive statistics of the sample

Demographics	Total sample (*N* = 14,740^1^) Frequency (%)
Gender, male	13,157 (89.3)
Age, years; mean (SD), [range]	24.1 (7.0) [14–75]
Age group; frequency (%)	
Age group 14–17	2,488 (16.9)
Age group 18–25	6,779 (46.0)
Age group 26 or older	5,473 (37.1)
Education (number of years completed), mean (SD)	13.0 (2.7)
Marital status	
Single	7,105 (48.3)
In a relationship but living separately	3,357 (22.8)
Married/living in a partnership	4,166 (28.3)
Divorced	75 (0.5)
Widowed	7 (0.0)
Study status (yes)	6,927 (47.0)
Working status	
Does not work	5,378 (36.5)
Has a full-time job	7,449 (50.5)
Has a part-time job	782 (5.3)
Works on an ad hoc basis	1,125 (7.6)
Average time spent gaming; mean (SD)	
Time spent gaming on weekdays (hours)	3.3 (2.1)
Time spent gaming on weekend days (hours)	5.6 (2.7)
Time spent gaming a week (hours)	27.6 (14.9)
Number of GD symptoms exhibited (APA framework)	
0	8,854 (60.1)
1	3,005 (20.4)
2	1,277 (8.7)
3	568 (3.9)
4	289 (2.0)
5	138 (0.9)
6	64 (0.4)
≥7 (7,8,9 GD scores merged)	61 (0.4)
Number of GD symptoms (WHO framework)	
0	11,807 (80.1)
1	1,613 (10.9)
2	604 (4.1)
3	186 (1.3)
4	60 (0.4)

*Note*: SD = standard deviation; GD = Gaming Disorder; APA = American Psychiatric Association; WHO = World Health Organization.

^1^ The mismatch between the total N (i.e., 14,740) and the total Ns reported for each variable is due to missing values. Percentages do not always add up to 100% due to rounding errors.

##### The World Health Organization's (WHO) framework

The WHO GD criteria encompass (i) impaired control over gaming, (ii) increasing priority over other activities, (iii) continuation or escalation of gaming despite negative consequences, and (iv) significant functional impairment evident for at least 12 months (World Health Organization, 2020). Given the similarity between the WHO and APA criteria, we adopted the IGDT-10 to assess GD symptoms according to the WHO framework (Horváth et al., 2022). Scores were computed by calculating the participants' responses for dichotomized IGDT-10 items 4, 5, and 6 and merged items 9 and 10. Scores were summed and ranged from 0 to 4, indicating the number of GD symptoms exhibited according to the WHO framework. For the boxplots, GD categories were calculated so that scores of 0–3 indicated no risk of GD, while, in accordance with the ICD-11 recommendation, scores of 4 indicated such a risk (World Health Organization, 2020). The composite reliability of the scale with the four binary items was ω = 0.83 in the present study.

#### Gaming-related variables

##### Time spent gaming

Participants were asked to answer two separate questions and specify how many hours they usually spend playing video games on (i) weekdays and (ii) weekend days, respectively. Values ranged between 0 and 12; if participants played more than 12 h a day, they were instructed to select 12. Responses were made to one decimal space. The two variables were combined ([hours spent on an average weekday X 5] + [hours spent on an average weekend day X 2]), and the average weekly time spent gaming was used in the analyses.

#### Variables representing psychological distress

##### Satisfaction with life

We used the Cantril-Ladder to assess general satisfaction with life (Cantril, 1965). In studies that use this ladder, the participants are asked to picture their life as a ladder. The top rung represents the best possible life, whereas the bottom rung represents the worst possible life. They then indicate where they see themselves standing. We replaced the ladder with 10 empty stars. We asked the participants to color in the stars, indicating how they felt about their lives. Ten filled-in stars meant they were delighted with their lives, whereas 10 empty starts meant they had the worst life possible. The scale is considered a valid and reliable measure of one's subjective satisfaction with life and one's well-being (Levin & Currie, 2014; Mazur, Szkultecka-Dębek, Dzielska, Drozd, & Małkowska-Szkutnik, 2018).

##### Depression symptoms

We used the short, 6-item version of the Center for Epidemiologic Studies' Depression Scale (CES-D; Radloff, 1977) to assess depressive symptomatology. This version was previously used in the European School's Survey Project on Alcohol and Other Drugs (ESPAD) international survey (Hibell et al., 2012). Participants were asked to think of the previous three months and indicate the degree to which they agreed with items such as ‘*I felt depressed*’ on a 4-point scale (1 = ‘almost none of the time’; 2 = sometimes; 3 = often; 4 = ‘almost all of the time’). Scores were summed and ranged from 6 to 24, with higher scores indicating more depression symptoms. Internal consistency (Cronbach's alpha) of the CES-D scale was 0.80 in the present study.

##### Perceived stress

We used two unreversed items from the short, 4-item Perceived Stress Scale (PSS; Cohen, 1986; Cohen & Williamson, 1988) to assess the degree to which the participants felt their lives were stressful. We adopted this approach because some researchers have suggested that the presence of reversed items might be a source of measurement error (Pederson et al., 2024; Swain, Weathers, & Niedrich, 2008). Participants were asked to think of the previous three months and answer questions such as ‘*How often have you felt that you were unable to control the important things in your life?* on a 5-point scale (1 = ‘never’; 2 = ‘almost never’; 3 = ‘sometimes’; 4 = ‘fairly often’; 5 = ‘very often’). Scores were summed and ranged from 2 to 10, with higher scores indicating more perceived stress. The internal consistency (Cronbach's alpha) of the two items was 0.76 in the present study.

### Statistical analysis

Descriptive statistics, independent sample *t*-tests (comparing risk groups with no risk groups), and one-way analyses of variance (ANOVAs) were conducted using IBM SPSS version 28, and results were presented in the form of figures with 95% confidence intervals. The independent variables were the number of GD symptoms exhibited, and the dependent variable was the average weekly time spent gaming. Games-Howell post-hoc tests were also conducted, and the results are provided in the supplementary materials.

To explore how average weekly time spent gaming relates to variables representing PD (i.e., satisfaction with life, depression symptoms, and perceived stress), we conducted a latent profile analysis (LPA). This is a person-oriented statistical procedure that classifies the participants in a study into homogeneous groups according to a set of item-response patterns (Collins & Lanza, 2010). Using Mplus statistical software (version 8), we identified different sets of latent profiles based on PD. To develop a satisfactory model consisting of a final number of profiles, we examined (i) information criteria such as the Akaike information criterion (AIC), Bayesian information criterion (BIC), and sample size adjusted BIC (SSA-BIC); (ii) the Lo-Mendell-Rubin Likelihood Ratio Test (LMR-LRT); (iii) and the Entropy index for classification accuracy. We compared the amount of average weekly time spent gaming and the number of GD symptoms with the identified LPA profiles using the Block-Croon-Hagenaars (BCH) method, which uses weights that reflect the measurement error of the latent class variable (Asparouhov & Muthén, 2014, 2019). The test statistic calculated for the BCH was the Wald chi-squared statistic (Wald χ2) (Asparouhov & Muthén, 2014, 2019).

### Transparency and openness

All data and the codes for the LPA are available at the open science framework: https://osf.io/56jkg/. All research materials are described in detail in the manuscript.

### Ethics

We conducted our study in accordance with the Helsinki Declaration. The study was approved by the Institutional Review Board of ELTE Eötvös Loránd University.

## Results

### Descriptive statistics

Table 1 lists the socio-demographic characteristics and average time spent gaming for the overall sample (*N* = 14,740).

### Comparing time spent gaming across groups of gamers reporting different numbers of gaming disorder symptoms

**Fig. 1.**
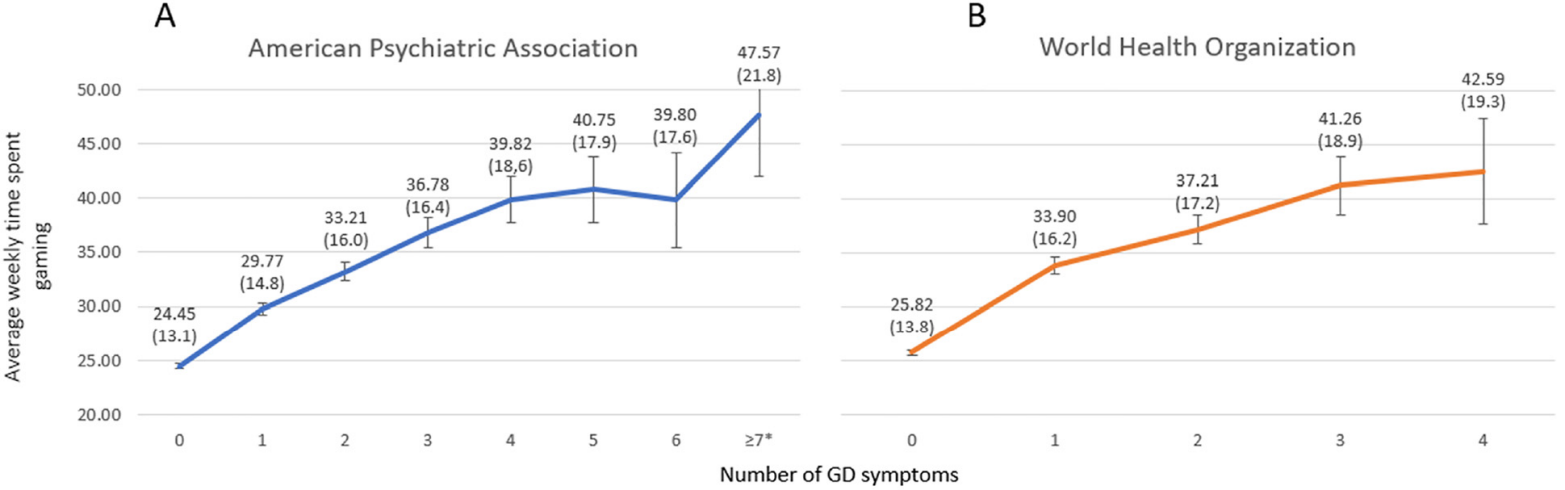
(A) Number of gaming disorder symptoms exhibited and average time spent gaming weekly (M; SD) according to the American Psychiatric Association's framework; *N* = 14,243 (B) Number of exhibited gaming disorder symptoms and average time spent gaming weekly (M; SD) according to the World Health Organization's framework; *N* = 14,257. Error bars represent 95% confidence intervals. *Note*: GD = Gaming Disorder; * = (7,8,9 GD scores merged)

One-way ANOVAs were conducted to explore the differences in average weekly time spent gaming between gamers exhibiting an increasing number of GD symptoms according to both the APA and WHO frameworks (see Fig. 1). To investigate the variation between the groups in detail and control for familywise errors, we conducted post-hoc tests (see Supplementary Tables S2.A and S2.B). In the case of the APA framework, there was a significant, positive, close to linear trend, indicating that as the number of GD symptoms increased, so did the average weekly time spent gaming (*F*(7, 14,235) = [208.224], *p* < 0.001, *η*^2^ = 0.09). Scores of seven, eight, and nine were merged because of the small sample sizes of these groups. The 95% confidence intervals revealed that there were no statistically significant differences (*p* ≥ 0.05) between those reporting five, six, seven, or more GD symptoms. According to the post-hoc tests, no significant differences were apparent between reporting three, four, five, or six symptoms or between six and seven or more symptoms (see Supplementary Table S2.A.). We assume that if the sample size of these groups (six GD symptoms and seven or more GD symptoms) were larger, the association with the average weekly time spent gaming would have followed a linear trend more closely, as was evident in the study by Pontes et al. (2022).

In the case of the WHO framework, a significant positive linear trend was also observed, indicating that as the number of GD symptoms increased, the average time spent gaming weekly increased (*F*(4, 14,252) = [249.619], *p* < 0.001, *η*^2^ = 0.06). No statistically significant differences – at the *p* < 0.05 level – were found between those exhibiting two, three, or four symptoms according to the 95% confidence intervals based on post-hoc tests (see Supplementary Table S2.B.). However, the lack of statistically significant differences is probably due to the small sample sizes of these groups.

**Fig. 2.**
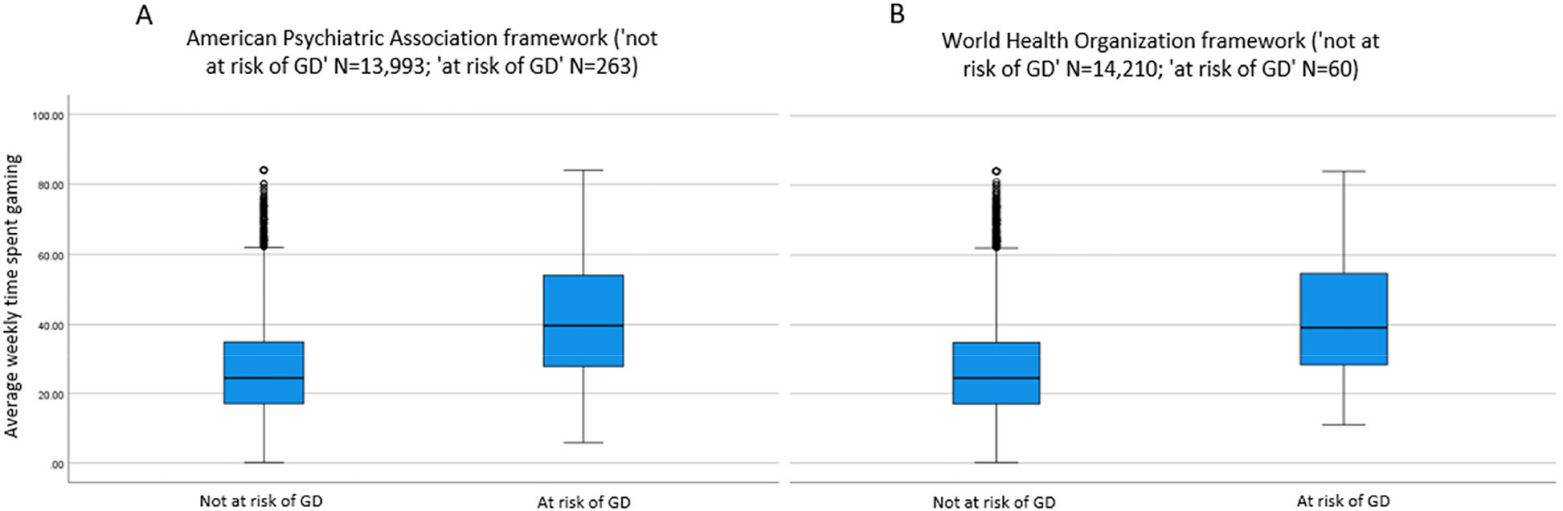
(A) Gaming disorder risk status and average time spent gaming weekly in hours according to the American Psychiatric Association's framework. (B) Gaming disorder risk status and average time spent gaming weekly in hours according to the World Health Organization's framework. The line across the boxplot indicates the median. *Note*: GD = Gaming Disorder

Figure 2 illustrates the differences in average time spent gaming weekly between those at risk of GD and those not at risk of GD according to the two frameworks. The mean weekly time spent gaming for those at risk of GD’ was 42.1 h (SD = 19.0) (Median = 39.7) and 42.6 h (SD = 19.3) (Median = 39.2), according to the APA and WHO frameworks, respectively. By contrast, those deemed not at risk of GD reported weekly time spent gaming of about 27.2 h (SD = 14.6) (Median = 24.4) (APA framework) and 27.4 h (SD = 14.8) (Median = 24.5) (WHO framework) hours on average. The differences were significant and large in both cases (APA framework: *t*(267.9) = −12.7, *p* < 0.001, *d* = 0.88; WHO framework: *t*(59.3) = −6.1, *p* < 0.001, *d* = 0.88).

### Comparing average weekly time spent gaming across groups of gamers with different psychological distress profiles

To identify sub-samples of gamers according to their level of PD, we conducted LPA of the participants' responses regarding their satisfaction with life, depression symptoms, and perceived stress. Table 2 lists the fit indices for the two- to five-profile models tested. The fit indices (i.e., AIC, BIC, and SSABIC) declined continuously as more profiles were added. However, the degree of decline diminished after the fourth latent profile was added. The Lo-Mendell-Rubin Likelihood Ratio Test (LMR-LRT) test evaluated the adjacent profiles and indicated that the four-profile model resulted in the most parsimonious solution.

**Table 2.** Fit indices for the latent profile analysis of the psychological distress variables (satisfaction with life, depression symptoms, perceived stress) (*N* = 12,943)

Model	Log-likelihood	Replicated log-likelihood	Nr. Of free parameters	AIC	BIC	SSABIC	Entropy	LMR-LRT test	*p*
2 profiles	−48529.789	Yes	10	97,079	97,154	97,122	0.821	9388.329	<0.001
3 profiles	−47298.226	Yes	14	94,624	94,729	94,684	0.75	2399.762	<0.001
**4 profiles**	**−46962.197**	**Yes**	**18**	**93,960**	**94,094**	**94,037**	**0.726**	**654.771**	**<0.001**
5 profiles	−46697.320	Yes	22	93,438	93,602	93,533	0.726	516.126	0.0954

*Note*: AIC = Akaike Information Criterion, BIC = Bayesian Information Criterion, SSABIC = Sample Size Adjusted BIC, LMR-LRT = Lo-Mendell-Rubin Likelihood Ratio Test. In bold: selected 4 profile solution based on fit indices.

**Fig. 3.**
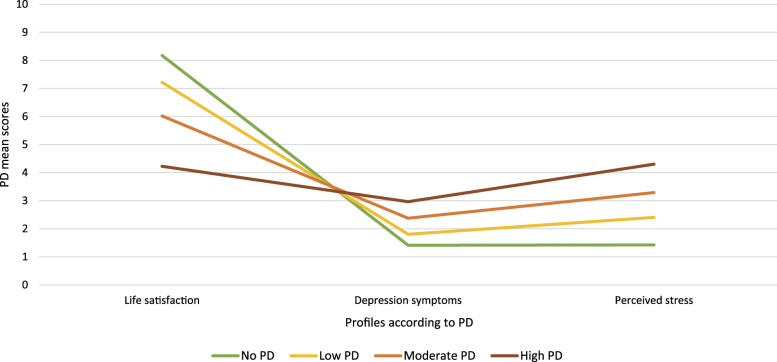
Latent profiles based on the latent profile analysis of the psychological distress variables (satisfaction with life, depression symptoms, perceived stress) (*N* = 12,943). *Note*: PD = psychological distress

Based on the information provided above, we chose the four profile solution and labeled each profile as follows: (1) no PD (*N* = 5,421; 41.9% of the sample), (2) low PD (*N* = 5,021; 38.8% of the sample), (3) moderate PD (*N* = 1,955; 15.1% of the sample), (4) high PD (*N* = 546; 4.2% of the sample). The labels mirror the extent of the overall PD reported (see Fig. 3).

Comparing the latent profiles identified, those in Profile 1, which comprises most of the sample (*N* = 5,421; 41.9%), had the highest level of satisfaction with life and did not experience depression symptoms or stress. Thus, they exhibited no signs of PD. Individuals in Profile 2, the second largest sub-group of the sample (*N* = 5,021; 38.8%), appeared to be less satisfied with life and occasionally had symptoms of depression and stress. Thus, these individuals might sometimes experience low levels of PD, which is neither considerable nor regular. Taken together, those in Profiles 1 and 2 have little or no PD. In contrast, those in Profile 3 (*N* = 1,955; 15.1%) reported substantially less satisfaction with life, coinciding with more depression symptoms and higher stress levels. Thus, those in Profile 3 are at risk of moderate and frequent PD. Lastly, individuals in Profile 4, the smallest sub-group of the sample (*N* = 546; 4.2%), had high PD, with the lowest levels of satisfaction with life, numerous symptoms of depression, and high levels of stress. On average, individuals in this group responded “often” (equals an average score of 2.965) to items in the depression measure, “fairly often” or “very often” (equals an average score of 4.3) to items in the stress measure and rated their satisfaction with life as 4 out of 10 stars on a scale where 10 stars indicate the best life and 0 stars the worst life. Therefore, based on their responses, those in Profile 4 experience considerable PD.

**Fig. 4.**
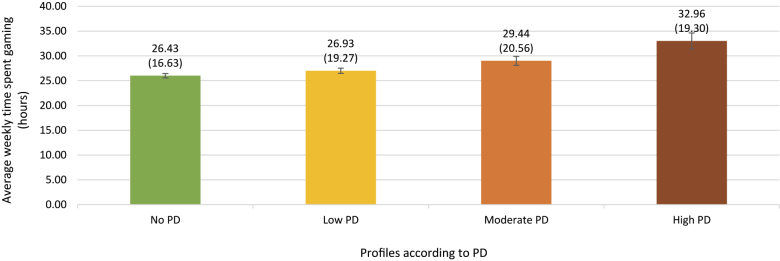
Average time spent gaming weekly in hours (M; SD) across all four latent profiles. Error bars represent 95% confidence intervals. *Note*: M = mean; SD = standard deviation; PD = psychological distress

We also compared the gaming-related variables, that is, average weekly time spent gaming and the number of GD symptoms according to both frameworks, across the identified profiles. The results in Supplementary Table S3 indicate that there were significant differences among the groups (Wald's test χ2(3) = 102.1; *p* < 0.001; *N* = 12,943) (Fig. 4; Supplementary Table S3). All profiles differed significantly from each other except for Profiles 1 and 2. Gamers belonging to Profile 4 with the highest levels of PD reported spending the most time gaming. Gamers who belonged to this profile played, on average, seven hours more a week than gamers in Profile 1 with no PD and at least four hours more than gamers in other profiles. Most notably, those in Profile 1, which constitutes 41.9% of the sample, reported no PD, even though playing for more than 26 h per week. Furthermore, although the difference between Profile 2 and Profile 3 in average weekly time spent gaming was significant, the magnitude (effect size) of this difference was small (Cohen's *d* = 0.10). In addition, the difference between Profile 2 and Profile 4 was also small (Cohen's *d* = 0.30), as was that between Profile 3 and Profile 4 (Cohen's *d* = 0.20). Supplementary Figure S1 provides the details about the distinction between the profiles according to the average time spent gaming on weekdays and weekend days.

## Discussion

We explored the association between the average weekly time spent gaming and GD symptoms according to the APA and WHO frameworks. Our goal was to replicate the study by Pontes et al. (2022) in an attempt to resolve some of the contradictory findings in the literature about this association. Furthermore, we tried to determine the amount of time that those who experience considerable PD, measured by their satisfaction with life, depression symptoms, and perceived stress, spend gaming.

We can make several observations by comparing our results with those of Pontes et al. (2022). According to their findings, the amount of gaming time associated with the risk of GD differed according to the diagnostic framework used (i.e., about 35 [APA] vs. 40 [WHO] hours/week). In our study, there was no considerable difference between the diagnostic frameworks, as the average time spent gaming for gamers at risk of GD was about 42 h per week (SD = 19), according to both. Furthermore, in both studies, GD symptoms gradually increased as gaming time increased. In our study, the increase was partially linear, but we assume that the reason was due to the small size of the samples exhibiting six and seven or more GD criteria. In the study by Pontes and colleagues, gamers who were not at risk reported no GD symptoms and played about 16–20 h per week on average, depending on gender and diagnostic framework. However, in our study, gamers not at risk played about 24–26 h on average, depending merely on the diagnostic framework.

One possible explanation for the higher numbers we found is the difference between the two data collection periods. While Pontes et al. analyzed pre-COVID-19 data, our information was collected during the first wave of the pandemic (March 2020), when people were isolated in their homes and admittedly spent more time playing video games (for more details on this point, see Supplemental Table S3 in Király et al., 2022b). Indeed, researchers have reported that levels of GD rose significantly during the pandemic (Rozgonjuk, Pontes, Schivinski, & Montag, 2022), as did self-reported time spent gaming (Barr & Copeland-Stewart, 2022).

To further determine how much gaming is too much, we explored how different average amounts of time spent gaming weekly are related to PD. We interpreted these differences among gamer groups using LPA analysis based on sample means. The results indicated that the group with the highest PD, who had the least satisfaction with life, numerous symptoms of depression, and high levels of perceived stress, reported playing on average for approximately 33 h per week. Thus, excessive gamers are prone to a greater risk of developing GD. Conversely, gamers unaffected by PD reported playing on average for approximately 26 h per week. This difference allowed us to infer that even a fairly large amount of gaming time can be a source of pleasure rather than a problem for most gamers.

Although our results imply that playing video games for more than 26 h a week seemingly puts players at risk of developing GD, we cannot determine a universal risk threshold of gaming time because it varies based on the players' personalities, psychological characteristics, and life circumstances (Griffiths, 2010). Furthermore, as the differences in the effect sizes among the four profiles were relatively small, our results should be used cautiously when considering practical steps to limit the time spent gaming. Moreover, our results pertain generally to older adolescents and young adults. Hence, additional research is warranted to investigate how much gaming time is too much for children and young adolescents. A fundamental step would be to explore how the gaming industry can help children learn how to manage their gaming time and encourage healthy playing habits without violating their rights and protecting their data. One example might be personalized warning messages to take breaks. While methods of restricting video game access or time might seem like a suitable option, children must be provided with opportunities to learn to regulate their behaviors (United Nations Children's Fund, 2019).

Our goal is not to determine the amount of time spent on gaming that is considered safe or unsafe. Rather, we provide data on the average amount of time spent gaming among different groups of gamers with varying levels of PD. In doing so, we highlight the various facets of video gaming as a healthy hobby, a life-enriching passion, or a problematic behavior. Reflecting on the results of our study, the time spent gaming does not appear to be a suitable or sufficient factor for determining the nature of the activity. This interpretation is also supported by previous literature in which the time spent gaming appeared to be a poor indicator of addiction (Billieux, Flayelle, Rumpf, & Stein, 2019). Furthermore, Huard Pelletier, Lessard, Piché, Tétreau, and Descarreaux (2020) showed that although increased gaming time was associated with indicators of poorer physical health, such as body mass index, the results depended on the type of gamer and the game, limiting any robust conclusions. Certainly, especially among individuals exhibiting problematic gaming behaviors, how their time spent gaming affects their social, educational, mental, and physical well-being could be a valuable avenue to explore. If mapped properly, the results could provide valuable insights that lead to helpful actions. Specifically, it is worthwhile examining under which circumstances prolonged gaming time occurs and how much in which condition is too much? For example, as was evident in our study, gaming activity on the weekend was longer than on weekdays (5.6 h per week versus 3.3, on average).

Another example is the motivation for playing video games. Do people spend more time playing video games because it is how they interact with meaningful others, master their gaming skills, or avoid everyday problems and alleviate their stress (Griffiths, 2010; Király et al., 2023)? Additionally, we must investigate the activities that people skimp on in order to play video games. For instance, are they skipping work or school to do so? Relatedly, when assessing the time spent gaming with self-report methods, a recent study proposed using the “Red Box, Green Box” method to differentiate between spare time (the “Green Box”) and times when the individual has responsibilities such as studying, working, sleeping, or exercising (the “Red Box”) (King, Billieux, & Delfabbro, 2024). Finally, we can also examine excessive consumption in a short period, namely binging, with regard to the scope of time spent gaming (Marmet et al., 2023).

Scholars have suggested that interventions focusing merely on limiting the amount of time spent gaming are insufficient if not combined with strategies addressing developmental and contextual elements (Throuvala, Griffiths, Rennoldson, & Kuss, 2021). To sufficiently limit the problematic use of video games, we must investigate other factors such as environmental, motivational, psychological, and neuro-psychological issues, particularly given their substantial contribution to GD (Horváth et al., 2022; Lee et al., 2018, 2021). Thus, despite its role as a potential predictor of GD, the amount of time spent gaming time is not a determinant of someone's developing such a disorder. Therefore, we suggest seeing gaming in a broader context and considering factors beyond the amount of time spent on video games that could determine whether it becomes a disorder, remains harmless, or is even advantageous.

### Strengths

Most studies examining the relationship between GD and time spent gaming usually include the latter as a potential risk factor along with other probable predictors. However, our study underscores how GD symptoms and different levels of PD align with specific values of the average weekly time spent gaming in a large sample of highly engaged gamers.

### Limitations

Despite our contributions to the literature, our study has several limitations that suggest promising avenues for future research. First, we used a cross-sectional design, which limited the exploration of causality. Therefore, our findings cannot inform the direction of the association between the average time spent gaming and PD. Second, we used convenience sampling, meaning that our sample contained an over-representative number of highly engaged gamers. This imbalance might have influenced the averages and characteristics of the various profiles. Similarly, Khazaal et al. (2014) showed that very engaged players of video games might be more likely to participate in online surveys related to gaming. Therefore, we suggest caution when interpreting our results and generalizing them to the broader gamer population.

Third, we used self-report measures. Thus, the reports the participants gave us about their average weekly time spent gaming might differ from the actual amounts. Additionally, the upper limit of gaming time per day was set to 12 h, and participants were instructed to choose this option if their play exceeded that amount. We did so to avoid unrealistic answers such as 24 h. However, as a side effect of this restriction, we were unable to determine the distribution of hours within this option. Future studies can use other, more objective measures of gaming time such as the diary method and tracking tools or different upper limits for daily gaming time to confirm the results. Fourth, we measured the variables associated with PD using shortened scales. Future studies can utilize more comprehensive assessments. Finally, our data collection period coincided with the first wave of the COVID-19 pandemic. In accordance with the “Green Box” notion, people spent more time at home during this period, which might have biased the results to a certain degree.

### Implications

The amount of time spent gaming appears to be linearly (or almost linearly) related to pathology. Parents and governments alike are concerned about those playing video games “too much.” However, opinions vary regarding how much gaming time is too much, with estimates being often overstated or underestimated. The present study aimed to help parents and policymakers address this question. While our results suggest that the amount of time spent gaming and the symptoms of GD are associated, spending extensive amounts of time gaming (up to 26 hours per week) did not appear problematic for most. However, longer amounts of time (up to 33 hours per week) are associated with various variables indicating PD as well as symptoms of GD.

Therefore, we would like to underscore the importance of the small-scope regulation of the time spent on gaming on a personal and familial level, one that considers individual characteristics and needs (Király et al., 2020). Larger-scope regulations, such as in-game notifications informing users how much time they have spent gaming, may also be useful (Király et al., 2018). However, we maintain that collective restrictions such as those imposed by the Chinese government might cause more harm (e.g., stigmatization; see, e.g., Ferguson, Bean, Nielsen, & Smyth, 2019) than good (Colder Carras et al., 2021; Király et al., 2022a). They could also prevent gamers from benefiting from the positive effects of judicious gaming (Johannes et al., 2021). Indeed, by limiting their autonomy in this regard, such regulations might also encourage illegal behavior designed to bypass them. To conclude, promoting awareness of and monitoring the time spent gaming is an indispensable tool in preventing GD and recovering from it (Gavriel-Fried et al., 2023). Nevertheless, it should be done sensibly and responsibly.

## Supplementary materials

## Data Availability

All data are available at the open science framework (OSF): https://osf.io/56jkg/
