# Supplementary material for: How much gaming is too much? An analysis based on psychological distress
Source: J Behav Addict. 2024 Jul 18;13(3):716–28. doi: 10.1556/2006.2024.00036 (PMC11457029; doi:10.1556/2006.2024.00036)
Supplement: Supplementary file 1 [file jba-13-716-s001.pdf]

Katz, D. et al.: How much gaming is too much? An analysis based on psychological distress

Supplementary materials

https://doi.org/10.1556/2006.2024.00036

Supplementary Table S1. Correlation between the three study variables: depression symptoms, perceived stress, and satisfaction with life

	Perceive stress	Depression symptoms	Life satisfaction
Depression symptoms	.649**		
Life satisfaction	-.476**	-.454**	
Average weekly gaming time h/w	.052**	.087**	-.132**

Note: **p < 0.01

Supplementary Table S2.A. Games-Howell post hoc group comparisons according to the American Psychiatric Association (APA) framework

Number of endorsed GD symptoms (APA)	Sample size of the subgroups	Time spent gaming (hours) Mean (SD)	Number of endorsed GD symptoms with significantly different ($p < 0.05$) mean of time spent gaming
0	8,854	24.45 (13.09)	1, 2, 3, 4, 5, 6, ≥ 7
1	3,005	29.77 (14.76)	0, 2, 3, 4, 5, 6, ≥ 7
2	1,277	33.21 (15.96)	0, 1, 3, 4, 5, 6, ≥ 7
3	568	36.78 (16.42)	0, 1, 2, ≥ 7
4	289	39.82 (18.57)	0, 1, 2, ≥ 7
5	138	40.75 (17.88)	0, 1, 2, ≥ 7
6	64	39.80 (17.62)	0, 1, 2
≥ 7	61	47.57 (21.82)	0, 1, 2, 3, 4, 5

Note: GD = gaming disorder; SD = standard deviation

Supplementary Table S2.B. Games-Howell post hoc group comparisons according to the World Health Organization (WHO) framework

Number of endorsed GD symptoms (WHO)	Sample size of the subgroups	Time spent gaming (hours) Mean (SD)	Number of endorsed GD symptoms with significantly different ($p < 0.05$) mean of time spent gaming
0	11,807	25.82 (13.77)	1, 2, 3, 4
1	1,613	33.90 (16.22)	0, 2, 3, 4
2	604	37.21 (17.20)	0, 1
3	186	41.26 (18.88)	0, 1
4	60	42.59 (19.28)	0, 1

Note: GD = gaming disorder; SD = standard deviation

Supplementary Table S3. Comparison of the four latent profiles in terms of gaming time variables

	Profile 1: No PD (n = 5,421) M (SD)	Profile 2: Low PD (n = 5,021) M (SD)	Profile 3: Moderate PD (n = 1,955) M (SD)	Profile 4: High PD (n = 546) M (SD)	Wald χ^2
Average time spent gaming on weekdays (hours)	3.14 (2.36)[a]	3.18 (2.76)[a]	3.52 (2.96)[b]	3.96 (2.80)[c]	79.75***
Average time spent gaming on weekend days (hours)	5.37 (3.16)[a]	5.51 (3.54)[a]	5.92 (3.62)[b]	6.58 (3.32)[c]	109.03***
Average weekly gaming time (hours)	26.43 (16.63)[a]	26.93 (19.27)[a]	29.44 (20.56)[b]	32.96 (19.3)[c]	102.08***
Gaming disorder symptoms according to the APA framework (range 0-9)	0.26 (0.88)[a]	0.67 (1.42)[b]	1.47 (1.94)[c]	2.44 (2.31)[d]	1649.09***
Gaming disorder symptoms according to the WHO framework (range 0-4)	0.00 (0.07)[a]	0.01 (0.14)[b]	0.04 (0.26)[c]	0.18 (0.42)[d]	178.14***

Note: M (SD) = mean and standard deviation; PD = psychological distress; APA = American Psychiatric Association framework; WHO = World Health Organization. The Block-Croon-Hagenaars (BCH) test was used for pairwise comparisons between the latent profiles. Wald χ^2 = Wald chi-square-test statistic for the overall difference between the profiles using the BCH method. Level of significance: ***p<.001. Different superscript letters (a, b, c, d) in the same row reflect significant ($p < 0.05$) differences between the means, while the same superscript letters in one row reflect a non-significant difference between the means, according to pairwise χ^2 equality tests of means across profiles using the BCH procedure with 4 degrees of freedom for the overall test for each variable compared.

Supplementary Figure S1. (A) Average weekday time spent gaming in hours (M; SD) across all four latent profiles. (B) Average weekend-day time spent gaming in hours (M; SD) across all four latent profiles. Error bars represent 95% confidence intervals.

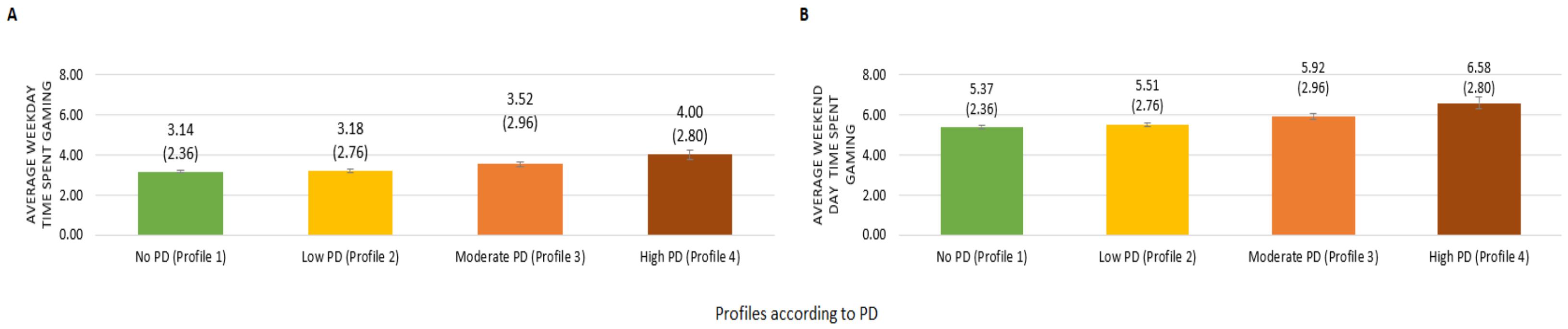

Note: PD = psychological distress
